# Resilience applications to social isolation and loneliness in older adults: a scoping review to develop a model and research agenda

**DOI:** 10.3389/fpubh.2025.1589781

**Published:** 2025-08-15

**Authors:** Andrew Wister, Boah Kim, Mélanie Levasseur, Valérie Poulin, Sarah Qiu, Esther Yuwono, Stéphanie Meynet, Julie Beadle, Laura Kadowaki, Katarzyna Klasa, Igor Linkov

**Affiliations:** ^1^Gerontology Research Centre and Department of Gerontology, Simon Fraser University, Vancouver, BC, Canada; ^2^Gerontology Research Centre, Simon Fraser University, Vancouver, BC, Canada; ^3^School of Rehabilitation, Faculty of Medicine and Health Sciences, Université de Sherbrooke, Sherbrooke, QC, Canada; ^4^Research Centre on Aging, Eastern Townships Integrated University Centre for Health and Social Services–Sherbrooke Hospital University Centre, Sherbrooke, QC, Canada; ^5^Department of Occupational Therapy, Université du Québec à Trois-Rivières, Trois-Rivières, QC, Canada; ^6^Gerontology Research Centre, Simon Fraser University, Vancouver, BC, Canada; ^7^Department of Gerontology, Simon Fraser University, Vancouver, BC, Canada; ^8^US Army Engineer Research and Development Center, Concord, MA, United States; ^9^University of Michigan School of Public Health, Ann Arbor, MI, United States; ^10^US Army Engineer Research and Development Center, Concord, MA, United States

**Keywords:** resilience, social isolation, loneliness, older adults, theoretical model

## Abstract

**Background:**

The development of a theoretical model applied to social isolation and loneliness (SI/L) among older adults has not kept pace with the exponential growth in empirical research, especially since the COVID-19 pandemic. One promising but under-investigated area is the contribution of resilience models to this field. This paper provides a scoping review of the application of resilience theoretical models to social isolation and loneliness and suggests directions for the development of an integrated new model.

**Method:**

Using the Arksey and O’Malley scoping review method, searches of four databases with 13 keywords were conducted April 9, 2024, with 17 articles meeting the inclusion criteria of the 1,671 extracted articles.

**Results:**

Findings were summarized using thematic analysis separated into four major themes: (1) coping self-efficacy to reduce SI/L; (2) moderating expectations to foster resilience to SI/L; (3) the effects of social support, the environment and resilience on COVID-19 stressors, and; (4) resilience as a mediator between SI/L and mental health. We integrate these findings into a new model entitled the Resilience and Social Isolation Model of Aging (RSIMA).

**Conclusion:**

RSIMA highlights SI/L as a dynamic process on a continuum, as well as elucidating what broader factors can lead to improved social connection, contributing to both individual-level and community resilience. To address the looming public health crisis of social isolation and loneliness among older people, future research studies must consider a systems-level perspective to SI/L and resilience.

## Background

Social isolation and loneliness (SI/L) have received increasing attention as important public health issues for older adults (1). Over the last two decades in particular, there has been growing alienation due to increased social media uptake coupled with major events such as COVID-19 pandemic policies that reduced social contact and fostered fears of social contact (2, 3). Social isolation is defined as the reduced quantity and quality of social relationships; whereas loneliness pertains to the perception that social needs are not being met (4). It is recognized that while social isolation and loneliness often occur together, they can be unique since a person can have many social contacts but feel lonely and vice versa (5). We distinguish social isolation and loneliness when used separately in research, but combine them to discuss overlapping conceptual or empirical findings. SI/L has been shown to double the risk of all-cause mortality in older adults (6, 7), and to a spectrum of physical and mental health outcomes (4, 8–10). Indeed, a recent report by the US Surgeon General warned that an epidemic of SI/L has been an overlooked public health crisis with the potential to significantly harm the health of the nation (11).

Prevalence rates of SI/L pre-pandemic have been estimated in many studies and countries with variations based on populations and research design. In a systematic review pre-pandemic, Chawla et al. (12) reviewed 31 studies globally and calculated an overall prevalence of loneliness of 28.5 per cent. These rates have been found to be higher peri-pandemic and post-pandemic. For instance, Canadian Longitudinal Study on Aging (CLSA) data estimated relative increases in loneliness during the pandemic ranging between 33 and 67 per cent depending on age/gender group (13). In 2022, the National Institutes on Aging (NIA) surveyed adults aged 50 and over and found that up to 58% have experienced some degree of loneliness and that 41% are at risk of social isolation (1). Overall, SI/L is a common experience among a significant proportion and number of older adults.

There have also been many studies that have identified numerous modifiable and non-modifiable risk factors for SI/L among older adults [e.g., (4, 14–17)]. This identification has also been extended to include the pandemic period [e.g., (18, 19)]. These risk factors fall into several demographic and socio-ecological domains, including environment (e.g., housing/living arrangement, geographic location, and access to technology), individual and social characteristics (e.g., ethnicity, immigrant status, and caregiver status), physical characteristics (e.g., functional mobility and multimorbidity), and psychological factors (e.g., mental health and well-being). For instance, SL/I has been linked to sex, gender, partnership status and living arrangement, especially those of advanced age who are unattached (single, widowed, and divorced) and living alone (4, 15, 17, 18, 20). Additionally, risk for SI/L is higher among individuals with low income, poverty, or living in economically deprived or rural/remote communities (19, 21) and ethnic and gender minority groups (including Indigenous elders, new immigrants, and LGBTQ2+; 102) (13, 22). People with sensory loss, multimorbidity or other physical health challenges (4, 19) or poor mental health conditions (depression, anxiety, psychoses, etc.); and caregivers (23, 24) are also at greater risk for SI/L.

A wide range of protective factors have also been linked to SI/L. Some of these include: having strong support networks, engaging in rewarding leisure pursuits, and participating socially (15, 25–27); living with others (28); having a positive or resilient attitude (29); and access to technologies, such as computers, tablets, and smartphones (30, 31). Taken together, the nexus of risk/protective factors likely work in tandem or culminate to influence SI/L. These associations are further complicated by the fact that there may be bidirectional patterns between risk/protective factors and SI/L.

The development of theoretical models to link and add coherence to the plethora of risk and protective factors has not kept pace with the often-siloed research studies. One promising area has been the theoretical development of resilience applied to aging contexts. These build on a strength-based approach to understanding the ways in which older individuals bounce back and sometimes exhibit growth, in the face of various adversities, such as natural disasters, loss of a spouse, multimorbidity, and pandemics (32).

Early research on resilience stemming from the early 2000s focused on psychological resilience in reaction to mental health and family adversities among children or young adults that allowed for positive adaptation and growth. Psychological resilience includes positive psychology, adaptation to stress, with the inclusion of concepts such as self-efficacy, mastery and coping processes (33–38). More recently, resilience models have been applied to a variety of vulnerable groups facing various adversities at the environmental, community, family and individual levels, including older adults (39–48, 103). Specifically, *social* resilience has incorporated elements of social participation, social isolation, and community belonging (27, 49, 50, 103).

One comprehensive interdisciplinary model is the Unified Model of Resilience and Aging (UMRA) which have been developed and applied to the pandemic context (48). Resilience models, like the UMRA, seek to understand how and why some individuals, families, and communities recover from adversity better than others. The UMRA integrates individual and system-level processes that occur over the life course, acknowledging that an adverse event (a pandemic, illness, personal loss) creates disruption that can be age and time-dependent. As such, resilience depends not only on the activation of internal resources (positive attitude) but also external resources (e.g., support from family, friends or organization), which can vary with age and other factors. It also includes four system-level (organizational) functions used by the US National Academy of Sciences Resilience Model (51, 52) which are central to the present study: (1) Planning for adverse events requiring reductions in risk in response to an identified threat, (2) Absorption of stressors and outcomes associated with an adversity which is necessary to initiate resilience through recovery and adaptation, (3) Recovery occurring through various forms of short and long-term strength-based resilience, and (4) Adaptation relating to changes in the system to promote future resilience. Together, these contexts are understood as manifested across the life courses of individuals. However, to our knowledge, none of these models have yet to be applied to SI/L. This scoping review aims to answer the following question: what are the major thematic theoretical developments that connect resilience and aging to SI/L? The review also intends to develop a new model of resilience and SI/L applied to aging and older adults, and offer a research agenda to guide future studies.

## Methods

### Search strategy

This scoping review used the methodology outlined by the Joanna Briggs Institute’s (JBI) Reviewer Manual (53), which provides an overview of scoping review methods (54) and highlights the most recent updates, primarily based on the Preferred Reporting Items for Systematic Reviews and Meta-Analyses for Scoping Reviews (PRISMA-ScR) (55). We used the Population-Concept-Context (PCC) framework to guide the scope and eligibility criteria of this review. The population was defined as older adults aged 50 or older. The concept focused on resilience models/frameworks related to SI/L; and the context included diverse care settings where older adults reside and receive care, especially related to resilience, coping strategies, and/or SI/L (a more detailed eligibility criteria is shown below). A specific three-step search strategy was employed. First, the initial search was conducted using four targeted academic online databases—APA PsycInfo, AgeLine, CINAHL Complete and MEDLINE. The following set of keywords was used: (“resilience” OR “strengths” OR “coping”) AND (“isolation” OR “social isolation” OR “loneliness”) AND (“older adults” OR “older persons” OR “older adults” OR “aging”) AND (“theory” OR “model” or “framework”). The full search strategy is presented in Appendix 1. To ensure enough articles, no limitation was placed on the publication date. After screening the title and abstract of retrieved studies, the second step entailed comprehensive research using identified keywords and index terms across all targeted databases. The third step included a hand search based on the reference list of identified articles to obtain additional sources. Also, we reviewed the reference lists of identified systematic review articles to supplement the initial electronic search to identify missing articles.

### Inclusion and exclusion criteria

To maximize theoretical developments in this area, the age criterion included older adults aged 50 or older. The specific inclusion criteria entailed: (1) studies with a focus on resilience models/frameworks or coping strategies applied to SI/L, (2) studies whose target population was older adults aged 50 or over, (3) empirical studies including quantitative, qualitative, mixed or multi-methods research, and (4) studies published in English. An article was excluded if it: (1) targeted pediatric, youth, or young adult populations, (2) did not focus on resilience or coping/adaptation conceptual or theoretical conceptual frameworks and SI/L, (3) did not focus on SI/L as a study outcome; gray area articles not published in peer-reviewed outlets; and (4) if the article was a systematic review/scoping review.

### Procedure

**Figure 1:**
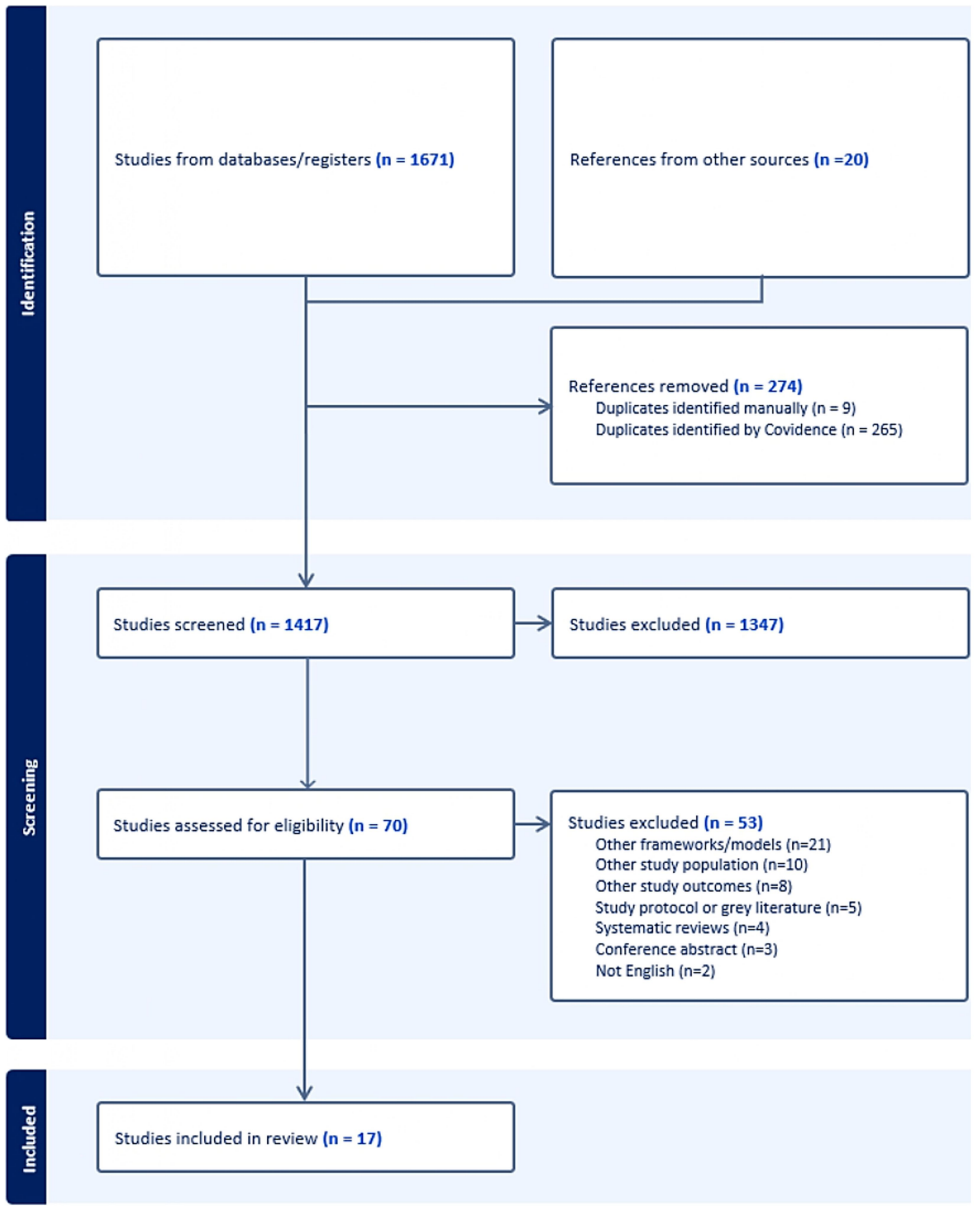
PRISMA flow diagram.

The screening procedure was conducted using the Covidence online platform^1^ (accessed on 9 April 2024). Covidence is a web-based systematic review program to streamline evidence synthesis process (56) and is well-suited for rigorous scoping reviews (57). Articles that met the inclusion criteria were imported to Covidence and duplicates were deleted. Two independent reviewers (BK, EY) completed two rounds of screening for the review. The first stage of screening entailed title and abstracts was conducted based on the eligibility criteria. Issues during this process were resolved based on the discussion with a third independent reviewer (AW). The second stage was a full-text review. A detailed screening process for exclusion is presented in a PRISMA flow diagram (Figure 1).

### Data analysis

The key characteristics of selected articles were extracted and organized by the main author into a spreadsheet. Data extraction included article identifiers (authors and year of publication), study details (population/sample size, country, objectives, design, data collection methods), type of adversity faced, and key findings. These data were crosschecked by other reviewers (AW, BK, ML, and VP) to discuss and adjust the detailed content. Thematic analysis following the approach proposed by Braun and Clarke (58, 101) was conducted by the same researchers to identify and synthetize the key themes across the selected studies. This method is useful in terms of systematically generating robust research findings by summarizing key features, patterns, common themes, and ideas within and across data, as it forces the researcher to take a well-structured approach to handle data in selected articles (59–62, 104). Using this approach, our study organized major themes to identify how resilience models/frameworks can be applied to address SI/L among older adults (Table 1).

**Table 1:** Characteristics of included studies.

Authors(s)/year	Study population/sample size	Country	Aims/Purpose	Type of adversity	Study design, data collection methods	Key findings
Akhter-Khan et al. (69)	NA	NA	To propose a theoretical model of social relationship expectations and loneliness	Loneliness in old age	Theoretical review	(1) Support in theoretical literature for a Social Relationship Expectation Framework Six Relationship Expectation Components: Availability of social contacts Receiving care and support Intimacy and understanding Enjoyment and sharing interest Generativity and contribution Being respected and valued (2) Context Note the importance of contextual factors: such as culture, functional limitations, social network changes
Boumans et al. (2)	Older adults aged 55 + in hospital setting (*N* = 231)	Netherlands	To develop program theories on coping in the face of Covid-19 isolation	Persons aged 55 + in hospitals during Covid-19	Qualitative- realistic evaluation	Both emotion-focused and problem-focused coping strategies were uncovered (1) Emotion-focused coping trust in staff facilitated by improved staff-patient relationships positive evaluation of conflict seeking support from family and friends & other patients acceptance and rationalization downward comparison (2) Problem-focused coping promoted by staff performing more person-centered cares open communication from the doctor to patients and to family & shared decision-making professional competency sense of control/mastery environmental context, such as single room
Chen et al. (75)	Older adults aged 75 and over (*N* = 1,646)	China	To explore the impact of social support and coping styles on depression and loneliness	Depression and loneliness	Quantitative, structural equation model of Baseline wave of China Longitudinal Aging Social Survey	(1) Social support is inversely associated with depression and loneliness (2) Social support is negatively associated with negative coping styles and predicts fewer symptoms of depression and loneliness (3) Social support is positively associated with positive coping styles and predicts fewer symptoms of depression, but not loneliness negative coping includes denial of problems, relying on others to solve a problem and accepting reality positive coping involves talking to others about the problem, changing behaviors, and learning from others to solve the problem
Gao et al. (78)	Older adults 60 and over (*N* = 4,531)	China	To examine the mediating effects of psychological resilience between hearing loss and social well-being	Older adults with hearing loss	Quantitative, multivariate analysis of the Sample Survey of Vulnerable Populations from Poor Families in Urban/Rural China, 2018	(1) Functional hearing loss (FHL) was negatively associated with social well-being (SWB) in low-income families, using social network and social participation scales (2) Psychological resilience using the Connor-Davidson 25-item Resilience Scale mediated 50.9% of the negative association between FHL and SWB resilience can reduce the negative effects FHL on SWB
Kastner et al. (76)	Older adults aged 50 and over (*N*=880)	Germany	To explore coping strategies among older adults to deal with stress during the COVID-19 pandemic	COVID-19 stress	Mixed-methods, qualitative content analyses to code text passages of coping strategies	(1) Three coping styles: problem-focused: active coping strategies such as structuring the day emotional-focused: regulating emotions such as keeping in touch with others, self-care, religion cognitive-focused: a reappraisal of the situation such as distraction or hope, (2) Personal prerequisites associated with coping value beliefs, living conditions (3) Problem-focused and emotional-focused were most relevant in coping (4) Lazarus and Folkman (77) stress transactional model used.
Ke et al. (106)	Older adults aged 60 and over from the first three waves of the US Health and Retirement Study, Baseline (*N* = 3,681)	USA	This study aimed to examine the long-term impact of social isolation on loneliness and depressive symptoms and to explore the moderating effect of resilience.	Depressive symptoms and loneliness	Quantitative analysis using cross-sectional analyses of Baseline wave and growth curve analyses of all three waves	(1) Based on the latent growth curve analyses, social isolation was significantly associated with more initial loneliness and depressive symptoms social isolation was associated with a slower increasing rate of loneliness, but no significant relationship with the change rate of depressive symptoms (2) Resilience significantly buffered the negative effect of social isolation on the initial level of depressive symptoms
Jackson et al. (40)	Older adults aged 65 and over from two studies; RUSH Memory and Aging Project (*N* = 782), Minority Aging Research Study (*N* = 28)	USA	To examine the effect of loneliness and change in loneliness on cognitive resilience – the discordance between actual and expected cognition given neuropathology	Neuropathology among older adults	Quantitative analysis of two longitudinal studies at Baseline and over time multivariate analyses	(1) Higher Baseline loneliness and change in loneliness over time was associated with lower cognitive resilience in the face of neuropathology emotional loneliness was found to be more important than objective levels of social contact issue of bidirectionality – cognitive decline may increase levels of loneliness
Lee et al. (63)	Older adults aged 65 and over (*N* = 159)	USA	To explore the association between coping self-efficacy (13 Item Coping Self-Efficacy Scale) and loneliness (UCLA 3 Item Scale dichotomized)	Older adults	Quantitative analyses – logistic regression	(1) Depression and social support were associated with coping-self efficacy (2) Loneliness was associated with coping self-efficacy, controlling for depression, social support, and other covariates coping self-efficacy may be one aspect of resilience
Lim et al. (41)	Older women aged 60 and over living alone (*N* = 308)	Korea	To investigate the mediating effects of physical health, resilience, and social support of loneliness and depression	Older women living alone	Quantitative analyses of cross-sectional data – multivariate mediation model	(1) Loneliness and depression were associated with physical health (self-rated health), resilience (Brief Resilience Coping Scale), and social support (Modified Medical Outcomes Study Social Support Scale) (2) Older women living alone with low levels of loneliness had reduced depression through high levels of resilience resilience mediates the association between loneliness and depression indicating the importance of psychological resilience on loneliness
Minahan et al. (74)	Adults aged 18–92 (*N* = 1,318)	USA	To examine (a) the effects of COVID-19 on depression, anxiety and loneliness; (b) the mediating role of coping style and social support; and (c) whether the above associations are age-related	Adults and older adults during COVID-19	Quantitative Analyses of cross-sectional data using path analyses	(1) Coping style has a mediating effect on the relationship between stress and depression, anxiety and loneliness during the COVID-19 pandemic, especially younger people (2) Avoidance coping style had the largest mediating effect on the relationship between stress and depression, anxiety and loneliness (3) Social support also had a mediating effect on these relationships older adults appeared to have greater resilience to stress during the COVID-19 pandemic compared to younger and middle-aged persons
Morgan and Burholt (68)	Older adults aged 65 and over (*N* = 11)	Wales	To explore the coping strategies and social comparisons of older persons experiencing loneliness	Lonely older adults	Qualitative interviews from the Wales Maintaining Function and Well-being in Later Life Study	(1) The degree to which older people view loneliness as modifiable affects experiences of loneliness (2) Problem, emotional, and meaning-focused coping styles appear to be interrelated and dynamic in their influence on loneliness experiences perceptions of loneliness are important for enhancing resilience to loneliness in older age
Morlett Paredes et al. (73)	Older adults living independently in senior housing (*N* = 30)	USA	To examine coping styles among older adults experiencing loneliness	Lonely older adults living independently in senior housing	Qualitative interviews	(1) Risk and protective factors: (a) age-related losses, lack of social skills, and protective attitude (wisdom, spirituality); (b) the degree to which older people view loneliness as modifiable affects experiences of loneliness (2) Sadness, lack of meaning and lack of motivation increase loneliness; and c) Coping strategies, such as acceptance, compassion, companionship, and environment stresses wisdom and social support, as well as emotional regulation, fosters greater resilience to loneliness
Ribeiro-Gonçalves et al. (79)	Older adults aged 60 and over (*N* = 349)	Portugal	To explore the mediating effect of resilience on the association between ageism, loneliness and psychological distress	Older adults aged 60 and over during COVID-19	Quantitative Analyses of cross-sectional data using path analyses	(1) Resilience (Connor-Davidson 10 Item Resilience Scale) fully mediated the association between ageism on psychological distress (Kessler Scale), and partially mediated the association between loneliness (UCLA Short Scale) and psychological distress. resilience protects against poor mental health from ageism and loneliness during COVID-19 income and education are associated with lower distress; and widowhood linked to high levels, controlling for covariates thus there is also a need to consider contextual factors
Schoenmakers et al. (66)	Older adults aged 62–100 (*N* = 1,187)	Netherlands	To investigate whether active coping (increasing relationships) or regulatory coping (changing expectations) affects coping with loenliness	OAs (65+)	Quantitative LISREL analyses of cross-sectional data from the 2010 wave of the Longitudinal Aging Study Amersterdam	(1) Active coping through vignettes was suggested less often to individuals who were older, in poor health, and lonely compared to mid-life working individuals (2) Regulatory coping suggested to people who were older, and for those who were older, with lower education and lower mastery the results suggest that lonely older adults should learn to adjust their expectations to realistic goals incongruence between expectations and actual experiences may be as important as simply increasing social networks
Van Baarsen (71)	Older bereaved adults aged 55–89 (*N* = 101)	Netherlands	To examine the role of self-esteem and social support in coping with bereavement. Also apply theories of coping with loss	Bereaved older adult	Quantitative-self-administered questionnaire	(1) Partner loss lowered self-esteem, resulting in more emotional and social loneliness (2) Supportive personal relationships lowered emotional loneliness (3) Friends increased both emotional and social loneliness over time neither theory supported need for new theoretical development
Warner et al. (44)	Older women aged 65 and over with at least one chronic illness (*N* = 138)	USA	To explore the effects of coping resources on loneliness and depression	Older women with chronic illness	Quantitative analyses using structural equation models	(1) Social support mediated the association between physical health and loneliness (2) Physical health affects depression through loneliness (3) Only greater social support was found to be a coping strategy for loneliness and depression (4) No support for religious coping and Selection, Optimization and Compensation model need for new theories of coping and loneliness, especially that incorporate physical health
Windle et al. (72)	Older persons aged 65 and over with cognitive impairment (*N* = 579)	Wales	To examine the effect of mental health resilience (no depression, no anxiety, high well-bring) on loneliness	Older people with cognitive impairment	Quantitative analyses using longitudinal (2 waves) of multivariate logistic cumulative effects model	**(**1) Mental health resilience predicted loneliness at time 2 (2) Social resources and higher self-esteem were associated with loneliness at time 2 supports a ‘resilience reserve’ hypothesis related to older persons with cognitive decline community supports are needed consistent with studies showing importance of positive attitude, higher self-efficacy, social support, physical activity, and esteem for reducing loneliness

## Results

### Description of studies

All 17 papers were published after 2001, with the majority (*n* = 15; 88.2%) in the last 5 years. Their studies were mainly conducted in Europe (*n* = 7; 41.2%) and North America (*n* = 6; 35.3%), but also in Asia (*n* = 3; 17.6%). Most (*n* = 12; 70.6%) used a quantitative design, three (17.6%) included qualitative methods, one (5.9%) a mixed-methods design, and one (5.9%) used a theoretical review. With sample sizes ranging from 11 to 4,531 older participants, eight studies (47%) focused on the general population, including one with adults aged 18 and over, and two (11.7%) specifically targeted women. The other studies each (5.9%) targeted: (*i*) widows, individuals with: (*ii*) chronic illness, (*iii*) hearing loss or (*iv*) cognitive impairment, or people living: (*v*) alone, (*vi*) in senior residences or (*vii*) hospitalized. While six studies (35.3%) investigated resilience in the context of loneliness, including two in the presence of depressive symptoms, three (17.6%) explored the strategies of older adults during the COVID-19 pandemic, and the other seven each focused on the specificity of their target population.

### Major thematic conceptual and theoretical developments that connect resilience and aging to social isolation and loneliness

Four themes provided unique resilience components and processes related to SI/L. These include: (1) Coping self-efficacy to reduce SI/L; (2) Moderating expectations to foster resilience to SI/L; (3) Effects of social support, the environment, and resilience on COVID-19 stressors and; (4) Resilience as a mediator between loneliness and aging-related challenges.

### Coping self-efficacy to reduce SI/L

Several studies provide theoretical and empirical evidence for the role of coping self-efficacy as a core concept in resilience processes linked to SI/L mitigation. Lee et al. (63) draw on Social Learning Theory developed by Bandura (64), and specifically its extension to coping self-efficacy, which is defined by the authors as “*self-confidence in one’s ability to effectively manage challenges using problem-solving, emotional regulation, and coping through social support.*” (p. 271). Using the 13-item Coping Self-efficacy Scale (65), the authors provide findings supporting an association between higher levels of problem-solving, emotional regulation, and social support subscales and lower levels of loneliness (63). Schoenmakers et al. (66) distinguish between two styles of coping: active coping which entails enhancing one’s social relationships, and regulative coping which involves lowering expectations pertaining to social relationships. Socio-emotional selectivity theory (67) posits that older people can employ several processes to lower expectations to foster higher levels of well-being. Schoenmakers et al. (66) build on Carstensen’s theory, specifically on the idea that, due to a reduced time perspective, older adults are more inclined toward regulatory adaptation. This involves prioritizing meaningful, high-quality relationships and activities over quantity, fostering immediate well-being and potentially reducing SI/L. Schoenmakers et al. (105) used four vignettes of loneliness experiences to identify their preferred coping styles and found that older adults with lower education and lower mastery more often used regulative coping than the other styles. Morgan & Burholt (68) also examine coping, finding that older adults who are confident that loneliness is modifiable are more likely to engage in a wide range of coping styles. These include problem-solving (e.g., enhancing social relationships), regulatory coping (e.g., lowering expectations of support), and meaning-focused coping (e.g., religiosity). The authors incorporate self-efficacy in terms of learning new coping skills through a computer education program, which was augmented through enhancement of social contacts with other mature students. Taken together, the authors (63, 66, 68) contend that these processes are not separate, but rather, comprise a dynamic process of resilience to reduce social isolation and loneliness.

### Modifying expectations to foster resilience to SI/L

A second major theme found in the articles of focus entails assessments and modifications to expectations (toward) and actual social relationships. In a theoretical paper that draws on psychology, gerontology and anthropology, Akhter-Khan et al. (69) develop the Social Relationship Expectation (SRE) framework to understand loneliness in old age from a life span sociocultural perspective. The six identified areas central to appraisals and actual social relationships include availability of contacts, receiving care and support, feeling close and understood, enjoyment and shared interests, generativity, and being respected and valued (69). The authors further contend that culture, functional status, and social network changes over the life course comprise contextual factors affecting relationship expectations among older adults and, ultimately, their loneliness. The primary coping strategies through which older adults adapt to relationship expectation incongruencies to maintain resilience is the Selective Optimization with Compensation (SOC) Model (70). Strategic selection, optimizing one’s available resources, and adapting to losses through compensation adaptation comprise the SOC Model. This model has been used to explain why some older adults during the pandemic adjusted to restricted social isolation by adjusting their expectations, and employing new strategies of communication to mitigate loneliness.

Other articles empirically tested theoretical concepts used to explain coping with loneliness. Focusing on partner loss among older adults, Van Baarsen (71) tests constructs derived from a general coping and specific theory of coping. The Theory of Mental Incongruity in which loneliness is the result of discrepancies between social relations desired and actual ones, contends that, at the general level, having higher self-esteem and social support results in better coping with loss. Similar to the SRE framework, the Theory of Relational Loneliness is a specific approach to loss and recovery that is understood as deficits in attachment, social integration and self-worth that affect identity formation as well as social and emotional loneliness. The equivocal results for both theories suggest a need to better integrate concepts into a more unified theory.

Jackson et al. (40) found that the incongruity between older adults’ actual and expected cognition, given their neuropathology, is deemed to represent cognitive resilience (CR). In other words, those showing cognitive reserve that exceeds their neurological assessments suggests a resilience to detriments linked to brain health. Using two longitudinal data sets, loneliness was associated with lower CR, suggesting that older adults with higher loneliness experienced steeper cognitive decline than expected (40), p. 944. Defining mental health resilience (MHR) as the absence of depression and anxiety and exhibiting high well-being, Windle et al. (72) found in longitudinal data that the odds of MHR were increased in males, and those having higher self-esteem, more social resources and fewer memory complaints. In a qualitative study of loneliness among older adults living in a senior housing community, Morlett Paredes et al. (73) found that aspects of wisdom, through pathways such as spirituality, emotional regulation, self-reflection, decisiveness and compassion, were protective against loneliness. Furthermore, the authors identify coping strategies to mitigate loneliness, including acceptance of aging, compassion, companionship and the role of environmental programming to cultivate meaning and positive relationships were important. In a related study, Morgan and Burholt (68) found that loneliness trajectories in old age are affected by social comparisons and whether or not loneliness is modifiable. Further, Minahan et al. (74) discovered that avoidant coping mediated the effects between pandemic-related stress and psychosocial outcomes, especially depression, although the findings for loneliness were weak. However, one study (75) did not find that positive coping styles affect loneliness, but these did reduce levels of depression in a Chinese sample.

This theme epitomizes several core social-psychological aspects of the resilience-SI/L connection. Enhanced self-esteem and social relationships, and the incongruence and realignment of deficits in social expectation relativity, appear to be core processes underlying this association. In addition, emotional regulation, SOC and wisdom are coping strategies of relevance.

### Effects of social support, the environment and resilience on COVID-19 stressors

Three studies specifically address coping styles among older adults to reduce loneliness during the COVID-19 pandemic (2, 74, 76). Boumans et al. (2) and Kastner et al. (76) draw on Lazarus and Folkman’s (77) Stress and Coping Model in which coping resources and styles are viewed as potentially buffering effects between stressors and mental health outcomes. These authors found that emotion-focused and problem-focused coping strategies were employed by older adults to support resilience to loneliness during the pandemic, especially pertaining to long-term care (LTC) where the disease risk was highest. In a qualitative study, Boumans et al. (2) support five emotion-focused coping strategies: trust in staff; positive appraisals and acceptance of the situation; seeking support from family, friends, and other patients; emotional regulation; and positive relative comparisons. Problem-focused coping strategies entail: training of staff to provide personalized care and having familiar staff, provision of information to the patient and family and shared decision-making enhancing sense of control, single room occupancy and personal items to enhance familiarity. In a quantitative study of older people living in the community during the pandemic (76), several coping styles associated with pandemic resilience were identified. Beliefs such as positive attitude, confidence and adaptability, spirituality, and prior experiences with being alone contributed to lower loneliness. Material, living and financial contexts were also important, such as living with others, good health care, better living and housing conditions and financial security, and higher functional mobility and transportation options. Kastner et al. (76) also supported the relevance of psycho-social evaluations of the stressful pandemic events that mitigate loneliness, such as positive appraisals of being alone and lower concerns for threatening aspects of Covid-19. Similar to the prior study, Kastner et al. (76) found that problem-focused approaches included social relationships and keeping busy. Emotion-focused strategies entailed maintaining social contacts, engaging in health behaviors, mindfulness, and spirituality among others. In a quantitative study of pandemic stress on mental health and loneliness and the mediating role of social support and coping style across age, Minahan et al., (74) found that reducing avoidance coping and enhancing social support lower the effect of pandemic stress on loneliness. Older adults reflected greater resilience than younger persons through more positive coping styles, providing support for a stress-coping model.

Coping styles, coupled with social, psychological and financial resources during the pandemic enhance resilience processes that reduced loneliness during the COVID-19 pandemic. This theme emphasizes the importance of linking system-level contexts of social isolation and resilience to adversities nested in the broader socio-ecological domains embedded within individuals, families, communities and the physical environments in which individuals live.

### Resilience as a mediator between SI/L and mental health

Several quantitative studies focus on mediation models of resilience on SI/L among older adults facing different types of adversity, including mental health challenges, hearing loss, living alone, ageism, and chronic illness. Ke et al. (106) found support for the mediating effect of resilience on the association between social isolation on depression. For instance, resilience was negatively associated with both the intercept and slope of loneliness (*B* = −1.15, *SE* = 0.05, *p* < 0.001; *B* = −0.22, *SE* = 0.03, *p* < 0.001); and depressive symptoms (*B* = −0.21, *SE* = 0.02, *p* < 0.001; *B* = −0.07, *SE* = 0.01, *p* < 0.001). Psychological resilience also mediated the relationship between hearing impairment and social well-being (social engagement and network characteristics) (78). In addition, Lim et al. (41) showed that physical health, resilience and social support mediated the association between loneliness and depression. Similarly, Warner et al. (44) and Chen et al. (75) showed that social support mediated the association between loneliness and depression, suggesting a resilience process among older adults. In a parallel study, Ribeiro-Gonçalves et al. (79) demonstrated that resilience also mediated the relationship between the effect of loneliness on psychological distress.

This theme positions resilience as an important mediator that can reduce the negative effect of SI/L on deleterious mental health outcomes among older people. Some studies utilize individual resilience measures, such as the Connor-Davidson Resilience Scale, whereas others used ecological resilience measures, and one employed social support as a proxy for resilience.

## Discussion

### Theoretical building blocks and gaps

**Figure 2:**
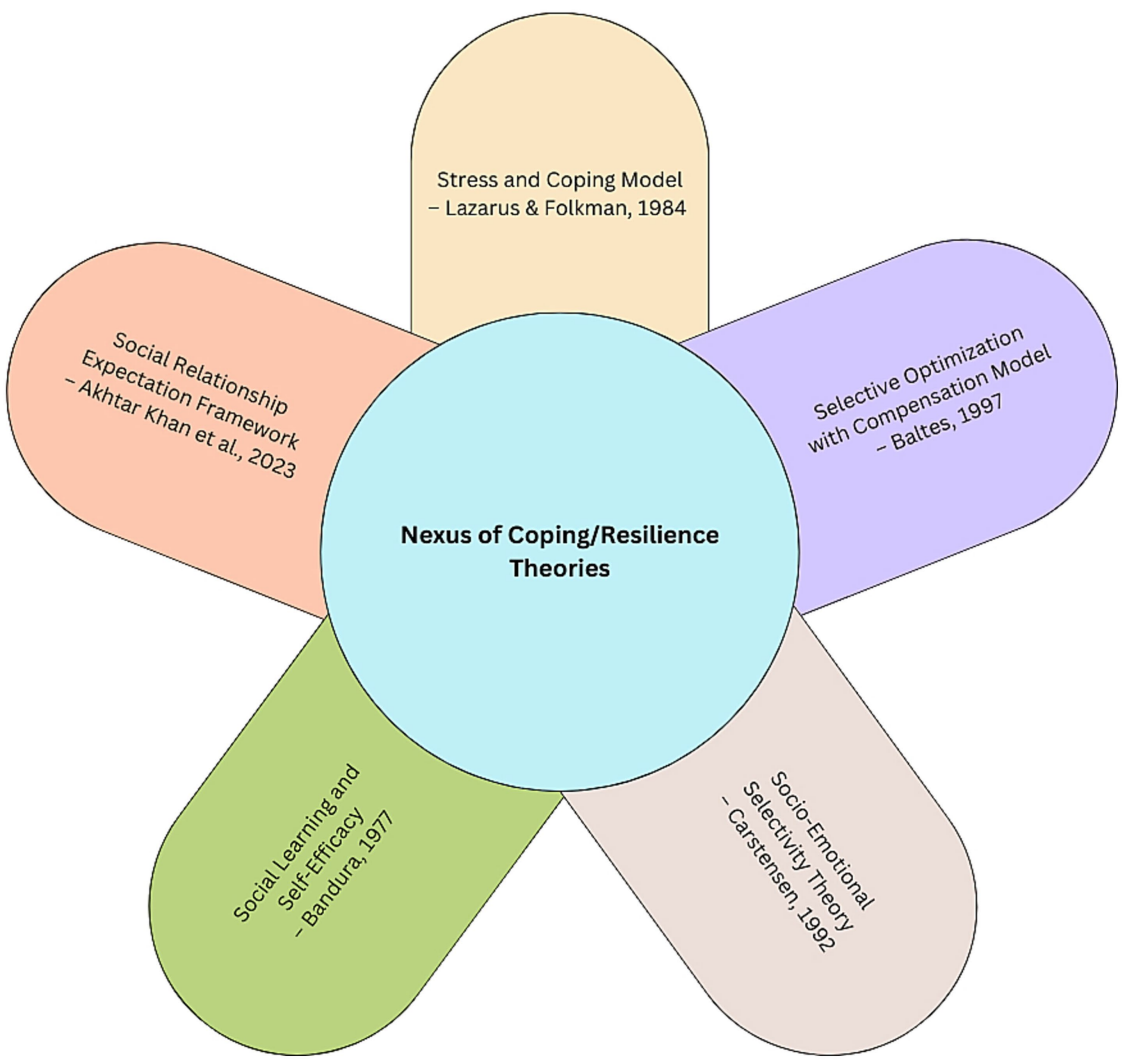
Coping and resilience theories applied to social isolation and loneliness.

This scoping review focuses on resilience and coping/adaptive theoretical applications applied to social isolation and loneliness among older adults. Taken together, the theoretical developments connecting resilience and SI/L have primarily drawn from psychological or social psychological approaches, including five primary theoretical models (see Figure 2). Akhter-Khan et al.’s (69) Social Relationship Expectation (SRE) framework; Lazarus and Folkman’s (77) Stress and Coping Model; Bandura’s (64) Social Learning Theory (especially self-efficacy concepts); Selective Optimization with Compensation Model (70); and Socio-emotional Selectivity Theory (67). In addition, empirically driven mediation models have also positioned resilience and social support as buffers between adversity stress and SI/L, either explicitly or implicitly framed using a Stress-Coping Model.

The conceptual and theoretical applications identify protective factors (e.g., strong self-efficacy, robust social relationships, positive attitude, etc.); coping strategies (e.g., problem-focused and emotional focused approaches; emotional regulation, expectation control, health behaviors, etc.); and environmental, financial and structural contexts (e.g., housing, LTC staffing, financial capital, etc.). While the themes overlap to some degree, they point to several central factors and processes that can be employed to enhance resilience to mitigate SI/L. Coping self-efficacy linked to both emotional and problem-solving is influenced by psycho-social attributes of the individual coupled with the role of social support. This core theoretical building block is important for the management of expectations. Additionally, the SRE emphasizes the regulation of expectations to align with social contexts in which older adults find themselves. Targeted selection and optimization with compensation where needed fosters greater adaptation to SI/L adversities. Thus, dealing with the incongruities of expected and actual social relationships and realignment of these manifests into improved resilience to SI/L. Stress and coping framing of SI/L points to the importance of buffering effects, which may include social support but also resilience facets. In addition, the pandemic-related stressors that exasperated SI/L were often structural in nature and point to the importance of moving beyond social-psychological resilience and coping to a system-level lens. For instance, the preparedness and adaptability of long-term care environments (e.g., personal protective equipment access and use, staff training, number of residents per room, and rapid response ability) during the COVID-19 lockdown phase directly affected the resilience processes of older people. All of these conceptual and theoretical developments occur against the backdrop of various social, environmental and physical contexts. In other words, it is understood that psycho-social coping and resilience attributes and processes can not be understood outside of the broader socio-ecological environments in which aging occurs.

## Limitations

Several limitations affect the integration and application of these conceptual developments. First, while we specify research findings focusing on social isolation, loneliness or both, and integrate the results into an SI/L combined model, there may be nuanced theoretical implications for each concept. Second, the majority fall within a specific discipline, especially psychology. There is a gap in unifying theoretical developments that occur within a primarily siloed disciplinary context. Third, the broader socio-ecological domains, such as age-friendly community contexts, rurality, and the physical environment, have not been centrally positioned, although some of these environmental contextual factors were incorporated into Kastner et al.’s (76) and Boumans et al.’s (2) articles. This could be due to the limited amount of public health literature on resilience and social isolation and loneliness. Fourth, there have been few attempts to connect the micro and macro environmental contexts of the nexus of resilience and SI/L, although pandemic research has given attention to system-level factors affecting SI/L. Fifth, resilience has been measured in several different ways (e.g., Connor-Davidson Resilience Measure, short resilience scale, proxy variables such as social support, etc.), making comparison between studies’ results difficult. Sixth, measures social isolation and loneliness also differ across studies, such that interpretations need to be made with care. Seventh, resilience and social isolation theoretical connections need to apply to a broad range of adversities, ranging from individual-level mental health and cognitive challenges to disaster research, such as pandemics, climate change adversities, and other catastrophes. Further specification of the model may be required in future research. Seventh, many studies reviewed included both depression and SI/L, which convoluted some of the theoretical interpretations. Eighth, the role of *prevention* to protect against adversity stressors (e.g., COVID-19 shutdowns), versus *active* resilience coping processes (e.g., realigning expected and actual levels of social connections during the pandemic) have not been clearly delineated in the literature. Finally, our scoping review itself is limited by the data used (e.g., these may have resulted in a bias toward social-psychological approaches to resilience and SI/L), the possibility of missing relevant articles in the search strategy, and our interpretation of the themes.

### Toward an integrated resilience and social isolation model of aging

The development of theoretical work in this field exposes an opportunity for an integrated theoretical model of resilience and SI/L. Currently in the literature on social isolation and loneliness, there has been an absence of an interdisciplinary model that bridges psychological, social, environmental and socio-ecological domains. We aim to fill this void by connecting the themes identified in this scoping review into an integrated resilience and SI/L model applied to aging.

One recent theoretical framework positioned within the resilience and aging literature that has the potential for application to SI/L is the Unified Model of Resilience and Aging (UMRA) (48). This interdisciplinary model was developed and applied to adversities created by the COVID-19 pandemic including SI/L, and can be applied to a range of adversities (48). The UMRA guides the understanding of why some individuals, families, and communities adapt, recover, and sometimes grow out of adversity, and the attributes and processes that facilitate positive responses. Given that this model integrates individual and system-level processes that occur over the life course, it also responds to the themes and gaps revealed in the scoping review.

**Figure 3:**
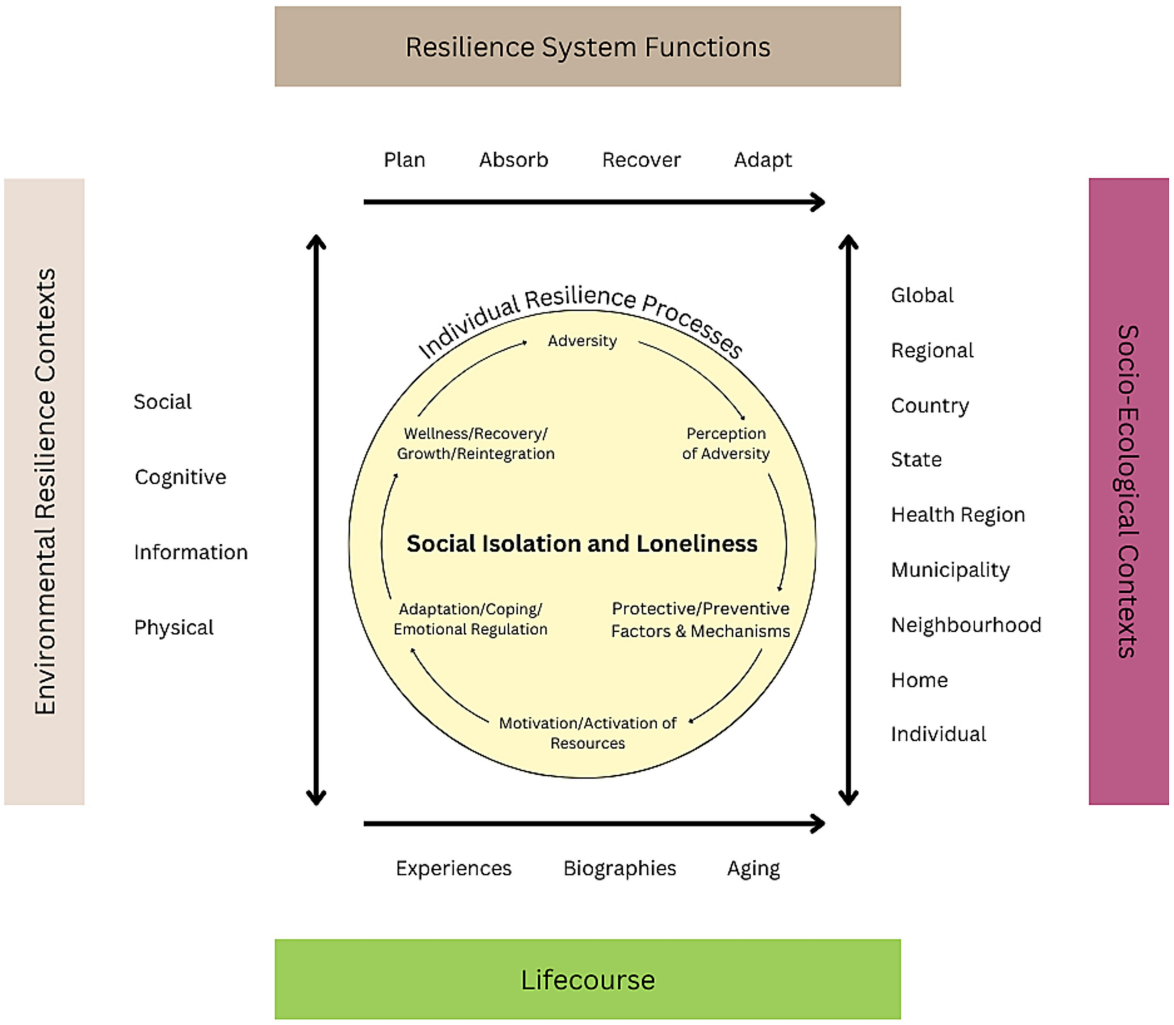
Resilience and social isolation model of aging.

Figure 3 presents an integrated model called the Resilience and Social Isolation Model of Aging (RSIMA), which is built on the scoping review and imputed into the UMRA. We use ‘social isolation’ in the title to capture both social isolation and loneliness. The primary difference between the UMRA and this proposed resilience model applied to SI/L entails the specification of the individual-level resilience processes based on this scoping review, and applying these to the original model. The central circle in Figure 3 of our proposed RSIMA model presents these individual resilience processes, some of which overlap the UMRA and three of which are unique. First, the RSIMA includes protective/preventive factors and mechanisms as a key component based on the scoping review results, in particular, the importance of social support. Second, we have incorporated emotional regulation with the adaptation and coping process consistent with our results depicted in Figure 2. Finally, RSIMA incorporates reintegration with wellness, recovery and growth to reflect the interconnections of these components as part of the final stages of the individual resilience processes.

The processes presented in the inner circle of Figure 3 demonstrate that, in response to SI/L adversity, resilience depends on (a) perceptions of the adversity (e.g., expectation realignment, severity, seriousness); (b) protective/preventive factors and behaviors (e.g., positive attitude, previous successful coping experiences); (c) potential disruption (e.g., disturbance to social roles, status, cognitive and physical health); (d) the activation of internal resources (e.g., self-efficacy, motivation to change attitude or behavior; and activation of external resources (e.g., buffering of stress and social support from family, friends, communities or organization, health behaviors); and (e) adaptation/coping/emotional regulation (emotional and problem-solving coping, selective optimization with compensation, buffering effects). The above processes culminate to affect wellness/recovery/growth, and reintegration).

The outer sections of the RSIMA model shown in Figure 3 borrows from the original UMRA model (48), which incorporates the primary system-level contexts within which individual processes are manifested (51). These align with the scoping themes pertaining to structural and socio-ecological contexts, especially related to, but not exclusive to, pandemic-related stressors that exasperated SI/L. These entail: (1) Resilience System Functions drawn from the US National Academy of Sciences Resilience formulation (plan, absorb, recover, and adapt) that was further codified in the US National Resilience Strategy (80); (2) the Socio-ecological spectrum from individual to global; (3) Environmental Resilience Contexts (social, cognitive, information and physical); and (4) Lifecourse temporal elements (experiences, biographies, aging). Specific to disaster research, including the recent COVID-19 pandemic, this resilience framing (51, 52) emphasizes: (1) Planning for adverse events requires reductions in risk in response to an identified threat, (2) Absorption of stressors and outcomes associated with adversity is necessary to initiate resilience through recovery and adaptation, (3) Recovery occurs through various forms of short and long-term strength-based resilience, and (4) Adaptation relates to changes in the system to promote future resilience. The coping processes are shaped by the socio-environmental contextual areas and the experiences, biographies, and aging processes embedded in the life courses of individuals.

Thus, this model highlights the dynamic multifaceted associations between social isolation, loneliness, and broader social determinants of health. It helps to frame our understanding of not only the causes of SI/L but also the effects of SI/L on the lives and experiences of aging and older adults. RSIMA emphasizes that individual-level resilience (i.e., psychological factors, physical health, life course changes, etc.) is intricately linked with community-level resilience (i.e., social factors of vulnerability, presence of robust safety net programs, etc.). Most existing models on resilience and SI/L do not use a systems-level approach. While individual-level factors are important in overcoming adversity, many broader upstream factors can have increasingly pronounced effects over the course of an individual’s life span (i.e., lack of health insurance or limited access to healthcare, poverty, non-English speaking populations, climate-induced changes to Indigenous peoples’ ways of life, etc.). Politics, programs, and greater global crises such as climate change are outside of a single person’s direct control, but they can directly impact both an individual’s level of resilience and a community’s ability to bounce back and adapt to any disruptions in their critical functioning (e.g., an area that burned down from wildfires).

#### Applications to social isolation and loneliness

The causes and consequences of SI/L can be understood from the RSIMA—a dynamic interdisciplinary integrative model that recognizes the socio-environmental and system-level contexts in which individuals engage in a variety of coping and adaptive resilience processes that are cumulative across the life course. These multilayered contexts comprising the RSIMA are specifically linked to the type, severity, and source of adversity, for instance, pandemic-related SI/L has unique properties compared to SI/L occurring during different periods or different contexts (e.g., individual, community, LTC).

The RSIMA framework adds to the understanding of theoretical and empirical work on SI/L applied to older adults in a number of significant ways. The expanding literature on social isolation and loneliness has been preoccupied with primarily identifying the risk and protective factors associated with SI/L, their consequences for health and well-being, as well as a variety of interventions [e.g., (8, 9, 17, 19, 81, 82)]. This is evidenced in over 30 systematic and scoping reviews pre-, peri-, and post COVID-19 pandemic [see (4, 13, 21, 83–85)]. However, much of this work has been empirically driven and typically has been located within disciplinary silos. The RSIMA offers an interdisciplinary, integrative framework with a resilience process lens applied to SI/L among older adults emphasizing a strength-based approach that can guide our understanding of the causes and consequences of SI/L and the development of practice and policy interventions. To this point, this model points toward the ways in which we can enhance coping processes to overcome the adversity embedded in SI/L.

Specifically, the RSIMA articulates protective and risk factors that reduce adversity associated with SI/L; reactive factors in response to adversity; activation of domain-specific resources; and adaptive processes in the formation of resilience. All of these lead to wellness, recovery, growth or reintegration in response to SI/L. Environmental risk factors, living alone, living in rural/remote areas, community and social deprivation, pandemic and other government policies, and other environmental adversities can increase the risk of SI/L among older adults (13, 72). Yet, these are interpreted as interacting with resilience strengthening protective and reactive factors, processes, and resources that can be activated, for instance, the existence or development of robust social support systems (71, 75), healthy lifestyles (48), and human and financial capital (79). Furthermore, these socio-environmental and resource-based domains shape and are interpreted through coping or adaptive social psychological resilience processes, such as realigning expectations against actual realities (2, 68, 76). For example, research suggests that older adults adapted well to isolating pandemic policies relative to other age groups since they adjusted expectations and drew upon life experiences of SI/L (74, 76). In this sense, the model provides a rationale for prevention, as well as proactive and reactive responses.

Furthermore, the RSIMA can be applied to this form of adversity to predict the potential outcomes of SI/L on older individuals, or the mediating or buffering effects of resilience on mental health, such as depression and anxiety (23, 41, 75, 86), and physical health, such as challenges to functional disability (4, 41, 87). In part, this is due to the dynamic reciprocal associations between SI/L and mental and physical health (19, 48). Additionally, as evidenced in the thematic analysis, the mediating role of resilience may interpret or buffer associations between risk factors, such as a functional hearing loss or cognitive decline, and SI/L (75, 78, 79, 106).

Finally, this model can also be potentially useful to analyze and guide the strategies that various organizations (e.g., community organizations, LCT, allied health and social services) use to adapt their services to promote the resilience of older adults in situations of SI/L or at risk of SI/L during and beyond a pandemic (88). Notably, this model can help different organizations visualize and understand the need for more interconnected services and improved collaboration and coordination across actors and organizations to meet not just individual needs but also population health needs for older adults.

For instance, hearing loss as a form of adversity has been associated with SI/L and cognitive decline (89); yet gaps remain in reducing the social, psychological, and physical effects of hearing loss. A resilience approach directs interventions to address this area through coordinated programs across multiple sectors (e.g., media campaigns, community services, government policies) at the individual, family, community, and system level. Based on RSIMA, these mechanisms can raise awareness, shift attitudes pertaining to hearing loss such as reduce stigma, enhance hearing testing and uptake of hearing aid use prior to deleterious effects on SI/L, and foster innovation in reaching hard-to-reach populations. Individuals can enhance their resilience to hearing loss effects on SI/L by also shifting their emotional regulation, attitudes and expectations, forms of individual adaptation and coping that are highly influenced by their social networks and support levels. Access to hearing aids, and the training and educational components needed for effective use, emphasize the salience of resource activation. Policy changes, such as health care insurance or policy changes to maximize coverage of hearing aids, represent the system-level of the model. These resilience factors and processes applied to hearing loss and cognition require future research to fine-tune the most effective mechanisms.

A major impediment to the development of programs and policies that aim to foster resilience and tackle SI/L are siloed and do not use a systems-level approach. With the growth in climate-driven crises (i.e., wildfires, heatwaves, avian flu) and the complex political challenges (i.e., migration, refugees, homelessness) that they create, strategies to bolster resilience in older adults will require a multi-sectoral approach that engages a broad group of stakeholders. Combating SI/Lin older populations will require strong collaborations between public health agencies and emergency management agencies, as well as private actors and non-governmental organizations.

### Formulating a theory-driven research agenda

Several research gaps can be identified and filled based on the RSIMA. First, comparative research needs to be conducted on different types, severity, and origins of SI/L. For example, current and future pre-pandemic, peri-pandemic, and post-pandemic SI/L adversity have both unique and shared elements. Pre-pandemic SI/L has been shown to have several predictors related to demographic, social, psychological, and environmental vectors often related to risk, marginalization, and vulnerability. Since pandemic-induced SI/L originated through government and public policy, there are likely nuanced experiences that may reveal new patterns of SI/L, as well as others that are similar to prior patterns but exacerbated. Post-pandemic SI/L may be a blending of the former types. More research that examines the effectiveness and the impacts of policies and programs that aim to increase resilience and decrease social isolation among older adults is needed. While resilience is a known concept among public health practitioners, it is a relatively new public health policy priority. Second, research is needed that employs an interdisciplinary lens to integrate the multilevel facets of SI/L stemming from this model. This requires data sets, in particular longitudinal data, that capture these contexts in conjunction with different designs and methods (e.g., multi-methods). Third, the assessment of risk for SI/L requires the development of indices that capture the different contexts in which it occurs, such as cultural, economic, and psychological ones (87, 90). One research gap is the investigation into inequality and diversity, such as gender, race, ethnicity, socio-economic status and other measures. Fourth, new data sources that contain complex multilevel measures (e.g., Artificial Intelligence to integrate multiple big data; national interdisciplinary longitudinal studies) are needed to reveal the complex interactions that are often overlooked in most statistical models. Fifth, although fixed variables are important in describing those at risk and forecasting trends (e.g., rates of living alone or singledom across age), there is a significant gap in knowledge about modifiable risk or protective factors, and how these factors are linked with higher level system components. Sixth, longitudinal interdisciplinary data are imperative to disentangle age, period, and cohort patterns in the predictors, contexts, and patterns of SI/L. For instance, shifts in systemic ageism in society over time may profoundly affect the prevalence and consequences of SI/L. Additionally, declining birth rates in some countries, particularly across Asia, are leading to a growing cohort of childless older peoples who will need to rely on non-traditional family structures and caregiving (91–93). Seventh, both basic and applied research are required to provide research evidence supporting this model, and the development, implementation, and evaluation of programs and interventions aimed at mitigating SI/L. Finally, policy research can assess the strengths and weaknesses of current approaches and point to the most promising and impactful innovations on the horizon. Our proposed and other theories and models of resilience across the life course need to be operationalized into actual policies and programs. Additionally, little is known about effective governance models that help support resilient communities. Future research should explore existing initiatives across urban and rural areas that aim to improve either resilience (overall) or social isolation in aging populations, identifying which actors are involved, what governance models look like, and whether these models promote a systems-level approach toward increasing resilience across the lifespan. Additionally, future research should also explore the role of multiple and/or cumulative crises on individual and community resilience. Short-term crisis governance can have important policy impacts that can either hinder or promote resilience as compound disasters increase in frequency and intensity over time (94).

## Conclusion

Neither Canada nor the United States have a comprehensive national policy that addresses social isolation and loneliness in older populations. While the US released its Federal Action Plan for Suicide Prevention in 2024 (95), SI/L is not directly addressed or discussed. In 2023, the US Surgeon General declared SI/L a key public health concern and released the first US national strategy to advance social connection (96). However, this strategy is yet to be operationalized and implemented across all US states. In 2024, the Canadian Coalition for Seniors’ Mental Health (CCSMH) established its first ever clinical guidelines on SI/L (81). However, similar guidelines for increasing social connection (and addressing SI/L) in older adults at a population health scale have yet to be created by public health agencies. SI/L is a growing public health concern with not only individual-level health consequences but broader societal impacts such as increased political polarization, decreased trust in institutions, and democratic backsliding or erosion.

The theoretical developments of the nexus of resilience and SI/L have grown rapidly in recent years. Understanding social connection—its structure, function, and quality—as well as the root causes of social isolation and loneliness are critical research priorities (96). SI/L are intricately linked to broader social determinants of health. This has been particularly apparent since the COVID-19 pandemic revealed significant individual and system-level gaps in knowledge, especially related to SI/L among older adults. These gaps in knowledge will only grow in importance as climate change and related crises grow, including the increased individual and societal risks related to compound disasters. For example, SI/L and social connection are found to contribute toward a community’s resilience (or lack thereof) to external crises such as natural disasters and other hazards (97–100). Most of the four themes that we identified focus on social psychological resilience and coping processes and mechanisms. There is a gap in existing research in exploring SI/L from a socio-ecological and system-level perspective.

The Resilience and Social Isolation Model of Aging (RSIMA) is an original theoretical framing that integrates interdisciplinary theories with a socio-ecological framing of resilience as a core component of social isolation, loneliness and social connectedness among older adults. RSIMA helps highlight how SI/L is a dynamic process on a continuum, as well as understand what broader factors can lead to improved social connection, contributing to both individual-level and community resilience. It is recognized that there have been public health interventions to address SI/L (e.g., UK Campaign to End Loneliness) that incorporate an integrative approach; however, many of these have not explicitly focused on older adults. Also, the limitations of this scoping review noted above warrant supplementary study in this field. Further empirical evidence, particularly using sophisticated systems-level analytic models in conjunction with social-psychological models, is needed to test the propositions drawn from this model. Future research studies must consider an integrated perspective to SI/L resilience to develop effective policies, programs, and interventions that address the public health crisis of social isolation and loneliness among older people.

## Data Availability

The original contributions presented in the study are included in the article/Supplementary material, further inquiries can be directed to the corresponding author.

## Footnotes

^1^ http://www.covidence.org/
