# Supplementary material for: Resilience applications to social isolation and loneliness in older adults: a scoping review to develop a model and research agenda
Source: Front Public Health. 2025 Aug 15;13:1589781. doi: 10.3389/fpubh.2025.1589781 (PMC12394976; doi:10.3389/fpubh.2025.1589781)
Supplement: Supplementary file 1 [file Table_1.docx]

**Supplementary material 1: Detailed Search Strategy**

**PyschINFO**

(“older adults” OR “older persons” OR elderly OR aging OR senior* OR gerontology* geriatr*) [tiab] AND (isola* OR “social isola*” OR lonel* OR “social exclusion” OR “social deprivation”) [tiab] AND (resili* OR strengths OR cooping OR hardiness OR adaptation OR readiness OR preparedness OR response OR recovery OR adjustment) [tiab] AND (theo* OR model* OR concept* OR frame*) [tiab]; Anytime-April 2024; English=316 results

**Ageline**

(isola* OR “social isola*” OR lonel* OR “social exclusion” OR “social deprivation”) [tiab] AND (resili* OR strengths OR cooping OR hardiness OR adaptation OR readiness OR preparedness OR response OR recovery OR adjustment) [tiab] AND (theo* OR model* OR concept* OR frame*) [tiab]; Anytime-April 2024; English= 189 results

**CINAHL**

MH aged OR TI (“older adults” OR “older persons” OR elderly OR aging OR senior* OR gerontology* geriatr*) OR AB (“older adults” OR “older persons” OR elderly OR aging OR senior* OR gerontology* geriatr*) AND TI (isola* OR “social isola*” OR lonel* OR “social exclusion” OR “social deprivation”) OR AB (isola* OR “social isola*” OR lonel* OR “social exclusion” OR “social deprivation”) AND TI (resili* OR strengths OR cooping OR hardiness OR adaptation OR readiness OR preparedness OR response OR recovery OR adjustment) OR AB (resili* OR strengths OR cooping OR hardiness OR adaptation OR readiness OR preparedness OR response OR recovery OR adjustment) AND TI (theo* OR model* OR concept* OR frame*) OR AB (theo* OR model* OR concept* OR frame*); Anytime-April 2024; English = 527 results

**MEDLINE**

MM aged [MeSH term] OR (“older adults” OR “older persons” OR elderly OR aging OR senior* OR gerontology* geriatr*).ti,ab. AND (isola* OR “social isola*” OR lonel* OR “social exclusion” OR “social deprivation”).ti,ab. AND (resili* OR strengths OR cooping OR hardiness OR adaptation OR readiness OR preparedness OR response OR recovery OR adjustment).ti,ab. AND (theo* OR model* OR concept* OR frame*).ti,ab; Anytime-April 2024; English= 639 results

**Description**

• Standard years: ~2024

• Date: April 9, 2024

• Number of articles found: 1,671

• Platform: EBSCO

• Databases: APA PsycInfo, AgeLine, CINAHL, MEDLINE

PsycInfo: 316

AgeLine: 189

CINAHL: 527

MEDLINE with Full text (EBSCO): 639
